# Physiological Changes in *Mesembryanthemum crystallinum* During the C_3_ to CAM Transition Induced by Salt Stress

**DOI:** 10.3389/fpls.2020.00283

**Published:** 2020-03-17

**Authors:** Qijie Guan, Bowen Tan, Theresa M. Kelley, Jingkui Tian, Sixue Chen

**Affiliations:** ^1^College of Biomedical Engineering and Instrument Science, Zhejiang University, Hangzhou, China; ^2^Department of Biology, Genetics Institute, Plant Molecular and Cellular Biology Program, University of Florida, Gainesville, FL, United States; ^3^Department of Biology, University of Florida, Gainesville, FL, United States; ^4^Key Laboratory for Biomedical Engineering of Ministry of Education, College of Biomedical Engineering and Instrument Science, Zhejiang University, Hangzhou, China; ^5^Zhejiang-Malaysia Joint Research Center for Traditional Medicine, Zhejiang University, Hangzhou, China; ^6^Proteomics and Mass Spectrometry, Interdisciplinary Center for Biotechnology Research, University of Florida, Gainesville, FL, United States

**Keywords:** *Mesembryanthemum crystallinum*, salt stress, photosynthesis and photorespiration, C_3_ to CAM transition, physiology

## Abstract

Salt stress impedes plant growth and development, and leads to yield loss. Recently, a halophyte species *Mesembryanthemum crystallinum* has become a model to study plant photosynthetic responses to salt stress. It has an adaptive mechanism of shifting from C_3_ photosynthesis to crassulacean acid metabolism (CAM) photosynthesis under stresses, which greatly enhances water usage efficiency and stress tolerance. In this study, we focused on investigating the morphological and physiological changes [e.g., leaf area, stomatal movement behavior, gas exchange, leaf succulence, and relative water content (RWC)] of *M. crystallinum* during the C_3_ to CAM photosynthetic transition under salt stress. Our results showed that in *M. crystallinum* seedlings, CAM photosynthesis was initiated after 6 days of salt treatment, the transition takes place within a 3-day period, and plants became mostly CAM in 2 weeks. This result defined the transition period of a facultative CAM plant, laid a foundation for future studies on identifying the molecular switches responsible for the transition from C_3_ to CAM, and contributed to the ultimate goal of engineering CAM characteristics into C_3_ crops.

## Introduction

*Mesembryanthemum crystallinum* can switch its photosynthetic system from C_3_ photosynthesis to crassulacean acid metabolism (CAM) under drought or salt conditions. CAM is a specialized mode of photosynthesis that has nocturnal fixation of atmospheric CO_2_ into organic acids (e.g., malic acid) by phosphoenolpyruvate carboxylase (PEPC), whereby the CO_2_-storing organic acids are remobilized and decarboxylated to provide CO_2_ for the Calvin cycle during the day (Winter et al., 2015). *M. crystallinum* is also known as a succulent plant with more succulence in leaves at adult and flowering stages than at juvenile stage (Adams et al., 1998). Because the CO_2_-storing organic acids are mainly stored in mesophyll cells, some degree of succulence is required for CAM to be efficient (Males, 2017). The succulence ensures independence from limited or unpredictable water supply after the juvenile growth phase. Over the years, *M. crystallinum* has become a favorite halophyte model for studying C_3_ and CAM due to its facultative capability under stress conditions (Vinocur and Altman, 2005; Winter and Holtum, 2014; Winter et al., 2015; Males and Griffiths, 2017).

Osmotic stress and ionic stress are two main challenges for plants growing under salinity (Munns and Tester, 2008). Mature *M. crystallinum* can survive high salt concentrations because of its ability to store water and its capacity of epidermal bladder cells (EBCs) to sequester up to 1 M sodium salt for adjusting the osmotic pressure (Bohnert and Cushman, 2000). Salt tolerance combined with the high water use efficiency (WUE) makes *M. crystallinum* an efficient CAM plant (Agarie et al., 2007). A comprehensive transcriptomic analysis of EBCs showed that V-type H^+^-ATPase (VHA) subunits were highly induced for vacuolar Na^+^ deposition in the EBCs (Oh et al., 2015).

Stomatal behavior is another specific feature of CAM plants. Stomatal movement is controlled by many different factors, including light, CO_2_ concentration, and circadian clock. In C_3_ plants, stomata open in the day and close in the night, causing much water loss through diurnal transpiration during C_3_ photosynthesis. Under mild stresses, plants reduce stomatal conductance, leading to decreases in CO_2_ intake and mesophyll photosynthesis (Chaves et al., 2009). In CAM plants, stomata are closed in the day and open at night to minimize water loss during PEPC-mediated CO_2_ fixation in the night. Then during the day, the CO_2_-storing organic acids are decarboxylated to provide high concentration of CO_2_ for C_3_ photosynthesis with closed stomata.

Differences in gene expression, protein, and metabolite levels, as well as phenotypic changes have been studied in C_3_ and CAM plants (Aragón et al., 2012; Cosentino et al., 2013; Abraham et al., 2016; Chiang et al., 2016). For example, weekly morphological and physiological changes in micropropagated pineapple under CAM-inducing conditions were characterized (Aragón et al., 2012). However, studies focusing on the physiological and molecular changes in the model plant *M. crystallinum* during the C_3_ to CAM transition in a short period have not been published. Here we report the morphological and physiological changes in *M. crystallinum* under salt-stress induced transition from C_3_ to CAM photosynthesis. By studying the short transition period, we were able to investigate mechanistic changes of photosynthesis, especially the regulatory triggers in facultative CAM plants. We observed that high salt concentration in the soil influences *M. crystallinum* phenotypic changes. In addition, stomatal movement combined with the leaf succulence assay during the transition period also gave us insight into how the facultative CAM plants reduce water loss, and how leaf succulence was developed to facilitate CAM photosynthesis.

## Materials and Methods

### Plant Growth and Salt Stress

*Mesembryanthemum crystallinum* seeds were provided by Professor John C. Cushman at the University of Nevada. *M. crystallinum* seeds were germinated and grown in a plant growth chamber at 26°C during the day and 18°C at night in a 12/12 h day/night cycle. Each seedling was grown in a 946 mL foam pot with Sungro Propagation Mix soil (SunGro Horticulture, MA, United States). Plants were watered three times a week with 50 mL 0.5× Hoagland’s solution (Hoagland and Arnon, 1950) for 28 days. Then plants in the control group were watered daily with 50 mL 0.5× Hoagland’s solution, while those in treatment group were watered with 50 mL 0.5× Hoagland’s solution containing 0.5 M NaCl following the protocol by Cushman et al. (1990). All the experiments were conducted with four biological replicates unless otherwise stated, with each individual plant being an independent biological replicate.

### Relative Water Content Analysis

To obtain fresh weight of leaves, the third and fourth leaf were detached and weighed. Similarly, fresh weight of the remnant shoot was weighed and fresh weight of the whole shoot was calculated by summing the weights of the leaves and remnant shoot. The leaves and remnant shoots were wrapped in aluminum foil, immersed immediately in a 4°C distilled water bath and soaked for 12 h. After 12 h, leaf surface was quickly blotted dry using paper towels and leaf turgid weight was measured. Then leaves were dried in an 80°C oven for 12 h, and leaf dry weight was measured. RWC was calculated by RWC = [(Fresh Weight − Dry Weight)/(Turgid Weight − Fresh weight)] ^∗^ 100% (González and Gonzalez-Vilar, 2001). Four biological replicates were used in both control and salt-treated groups.

### Leaf Area Measurement

A python program was created using the ratio of green pixels against 1 cm^2^ black spot pixels used for measuring leaf surface area with images taken by a Cannon Rebel T6 DSLR camera (Supplementary Figure S1 and Supplementary File S1). Eight plants were divided into control and salt-treatment groups. They were photographed at 12 pm every day to track leaf growth, and the python program was used for leaf area measurement. Leaf area simulation was based on a model reported by Xiao et al. (1988), and parameters were calculated using minimum distances between simulation and acquired data.

### Gas Exchange Measurement

Gas exchange measurements were conducted by a portable photosynthesis system (Li-Cor 6800, Li-Cor Inc., Lincoln, NE, United States) equipped with a 68H-581066 fluorescence head and a 6 cm^2^ chamber. An air flow ratio was set to 800 μmol s^–1^ with chamber delta P at 0.2 kPa, and a fan speed of 10,000 min^–1^. Relative humidity in the chamber was set to 50% to be consistent with the humidity setting in the plant growth chamber, and the reference cell CO_2_ concentration was set to 400 μmol mol^–1^ air. Light intensity in the fluorescence head was set to track the light intensity measured by the external quantum sensor. A program was made to log parameters every 30 s and to match IRGA (infrared gas analyzer) every 30 min. The Li-Cor instrument was kept in the growth chamber for diurnal and nocturnal gas exchange monitoring. Three separate biological replicates were conducted for the gas exchange measurement.

### Stomatal Phenotype Observation and Measurement

A stomata tape-peel method (Lawrence et al., 2018) was used for stomatal movement observation. A piece of transparent adhesive tape was attached to the central part of the abaxial side of the third or fourth leaf, and a razor blade was applied to scrape away non-adherent cells. The tape with a thin layer of cells was put onto a microscopy slide, and then observed under a Leica DM6000 B microscope. Fifteen images of three biological replicates were taken in Openlab 5.5.3 in RGB mode for each group at each observation time point. Diurnal and nocturnal behaviors of stomatal movement were observed at 4 pm and 4 am, respectively. ImageJ was used to analyze microscopic images^1^ and150 stomata were observed from the 15 images. The stomatal aperture was measured by the width of inner pore, and the proportion of open stomata was calculated by division of the number of open stomata by that of total stomata (Eisele et al., 2016).

### Leaf Succulence Measurement

Leaf succulence was measured daily at 4 am for 14 days after start of control and salt treatment. Then the second pair of leaves from one plant was used as one biological replicate, and a total of four plants were measured. The leaf area was measured using the same protocol as described above using the python program, and leaf fresh weight was measured immediately after the leaves were excised. Leaf succulence (g cm^–2^) was calculated by leaf fresh weight/leaf area. Four biological replicates were used at each time point for each treatment.

### Malondialdehyde Content Measurement

We adapted a method from Wang et al. (2019) for measuring the malondialdehyde (MDA) content. Leaves of ice plants were harvested before and after salt treatment, and then ground to a fine powder in liquid nitrogen. A total of 3 mL 10% trichloroacetic acid (TCA) was added to 0.2 g leaf tissue powder and kept in 4°C overnight. After centrifugation at 10,000 × *g*, 4°C for 20 min, the supernatant (2 mL) was transferred to a new tube. Then, 2 mL 0.6% thiobarbituric acid was added to the supernatant, and vortexed thoroughly. The mixture was heated in boiling water for 15 min, cooled to 4°C immediately and centrifuged at 10,000 × *g*, 4°C for 10 min. Absorbance of the supernatant was recorded at wavelengths of 532 and 450 nm. The MDA content (μmol L^–1^) was calculated by 6.45 ^∗^ OD_532_ – 0.56 ^∗^ OD_450_. Four biological replicates were conducted at each time point.

### Statistical Analysis

Experimental values were processed using Numpy module and Scipy module installed on Python 3.5. Bars in the figures correspond to standard errors, and a star in the figures indicates *p*-value <0.05 based on Student’s *t*-test, and two stars indicate *p*-value <0.01.

## Results

### Plant Growth and Leaf Area Changes

**FIGURE 1:**
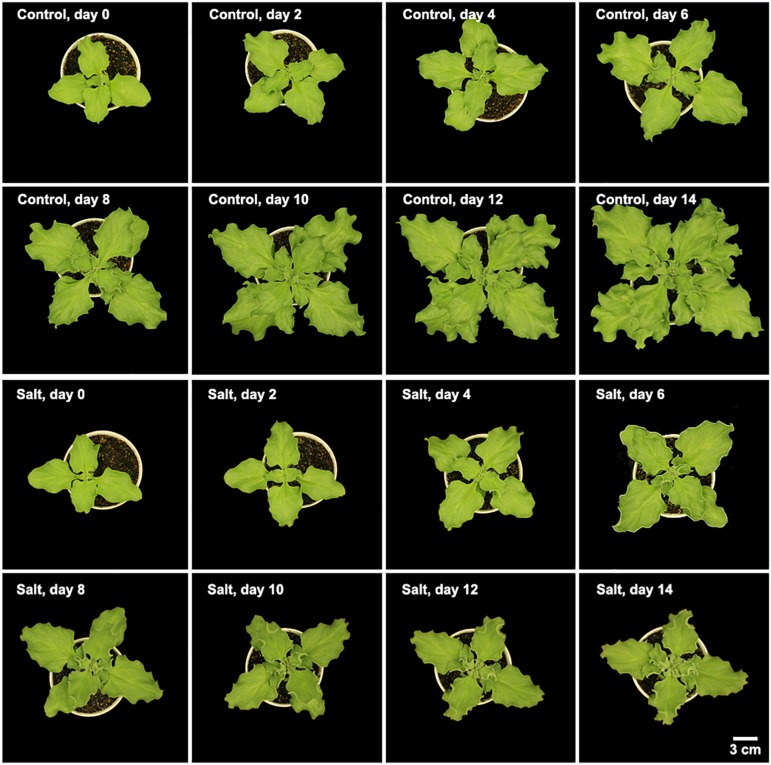
Morphology of *M. crystallinum* seedlings growing under control and salt treatment for 14 days. Each image is representative of the seedlings at 12 pm in the four biological replicates.

**FIGURE 2:**
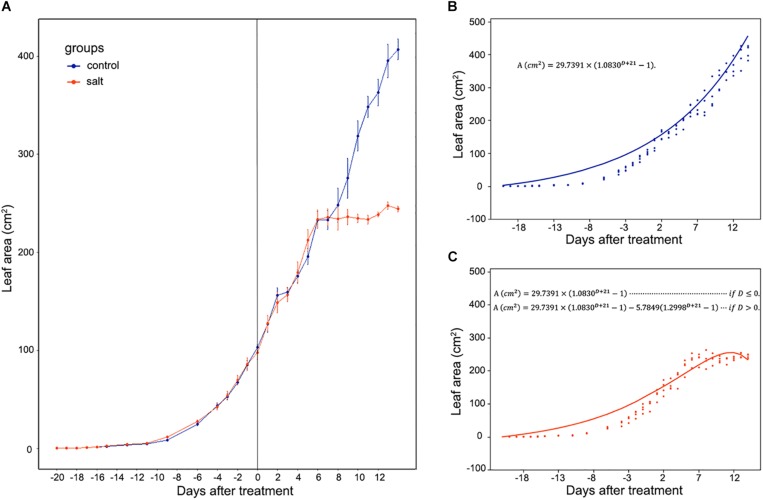
Whole leaf area measurement of *M. crystallinum* seedlings during their growth for 42 days. The salt treatment began at day 0. **(A)** Leaf area changes in control group (blue line) and salt-treated group (orange line) of *M. crystallinum*. **(B)** Simulation of *M. crystallinum* growth of the control group. The line represents the simulation and the dots represent exact leaf area of each biological replicate. **(C)** Simulation of *M. crystallinum* growth of the salt treated group. The line represents the simulation and the dots represent exact leaf area of each biological replicate.

*Mesembryanthemum crystallinum* seedlings have different leaf shapes at different developmental stages. In this study, we used seedlings at the early developmental stage (Figure 1) before they shift into CAM photosynthesis. After 4 weeks of growth, the second pair of primary leaves became as large as the first pair of primary leaves. Then we applied salt treatment to one group of the plants. In the first week of treatment, there are no significant plant growth differences between the control and salt-treated groups. After 1 week of salt-treatment, the treated plants decreased the rate of leaf growth compared to the control plants (Figure 1). Leaf area change is closely related to plant growth, development, and water usage. Here we developed a python script (Supplementary File S1) using leaf images with a 1 cm^2^ reference square on the same plane to accurately measure the leaf areas of *M. crystallinum* seedlings every day for a total of 56 days. Statistics analysis was performed between the leaf areas from control and treatment groups. Clearly, *M. crystallinum* seedlings had a fast growing trend (Figure 2) in the control group using a simulated equation: *A*(*cm*^2^) = 29.7391×(1.0830^*D* + 21^−1). *A* is the total leaf area and *D* is the days after treatment. After salt treatment, *M. crystallinum* leaves grew at a similar rate as the control group in the first 5 days, and then slowed down the growth after 7 days of salt-treatment with a simulated equation:

$$A\,\,\left({\text{cm}}^{2}\right)=29.7391\times \left(1.0830^{D+21}-1\right)\;\text{if}\,D\leq 0.$$

$$A\,\,\left({\text{cm}}^{2}\right)=29.7391\times \left(1.0830^{D+21}-1\right)-5.7849\,\left(1.2998^{D+21}-1\right)$$

if⁢D>0.

Since carbon fixation plays an important role in leaf expansion, these results indicate that after 7 days of salt stress, the ice plants may have less carbon fixation compared to the C_3_ plants.

### CO_2_ Assimilation and Transpiration

**FIGURE 3:**
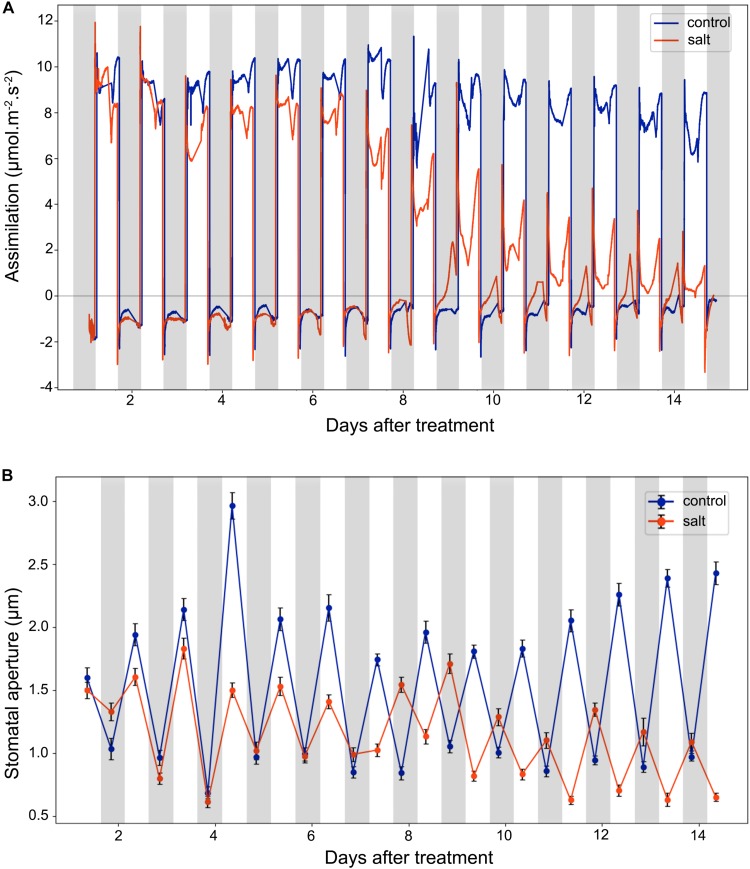
Leaf CO_2_ assimilation and stomatal aperture changes in the control and salt-treated *M. crystallinum* seedlings. **(A)** Leaf CO_2_ gas exchange and **(B)** stomatal movement behavior. The blue line represents the control group and the orange line represents the salt-treated group. The gray bars represent the night time and the white bars represent the day time. The error bar represents standard error. The data were obtained from three biological replicates.

Stomata conductance to water vapor is affected by many factors, such as stomatal aperture, CO_2_ concentration, light intensity, and temperature. In this study, we did gas exchange measurements for leaf assimilation rate continuously over 14 days after the salt treatment (Figure 3A). In control plants, the assimilation rate during the day is in the range of 6.0–12.0 μmol m^–2^ s^–1^, and in the range of −2.0 to 0.0 μmol m^–2^ s^–1^ during the night. In contrast, in the salt-treated plants, the assimilation rate has a decreasing trend in the day and an increasing trend in the night. The gas exchange result of control plants indicates that under well-watered conditions, the 42-day-old plants did not switch to CAM photosynthesis and the photosynthetic activity was steady in the leaves. The salt treatment results showed that the assimilation rate was decreased during the day after 6 days of salt-treatment. However, the assimilation rate during the night was increased after 6 days of treatment. After 8 days of the salt-treatment, the assimilation rate during the night was increase to almost 0.0 μmol m^–2^ s^–1^, indicating that switching to CAM took place between 6 and 8 days after treatment.

After 9 days of salt treatment, the plants had an interesting pattern of gas exchange rate, the lowest assimilation rate during the day was lower than 0.0 μmol m^–2^ s^–1^ at 2 pm, and the highest assimilation rate during the night was higher than 0.0 μmol m^–2^ s^–1^ at 2 am. When the chamber light initially came on, the assimilation rate culminated from lower than −2.0 μmol m^–2^ s^–1^ to >2.0 μmol m^–2^ s^–1^, and when the chamber light switched off, the assimilation rate dropped from higher than 1.0 μmol m^–2^ s^–1^ to lower than −2.0 μmol m^–2^ s^–1^ (Figure 3A). These results indicated the existence of the CAM circadian clock, which controlled stomata closure before the chamber light switched on and stomatal opening before the chamber light switched off.

### Diurnal Stomatal Movement

We measured diurnal stomatal movement patterns through analyzing stomatal aperture and the proportion of opening stomata every 12 h during the 14-day period after the salt treatment. As shown in Figure 3B, the size of *M. crystallinum* stomatal aperture was in a range of 1.7–3.0 μm while stomata were open, and at a range of 0.7–1.1 μm while stomata were closed. The inversed stomatal movement behavior in the salt-treated plants was observed between 6 and 7 days after the treatment, indicating that CAM photosynthetic mechanism starts to play a role at day 7. It was noticed that the overall stomatal aperture of salt-treated plants became smaller after 10 days of treatment, so did the size of the stomatal guard cells (Supplementary Figure S2).

### Leaf Succulence and RWC

**FIGURE 4:**
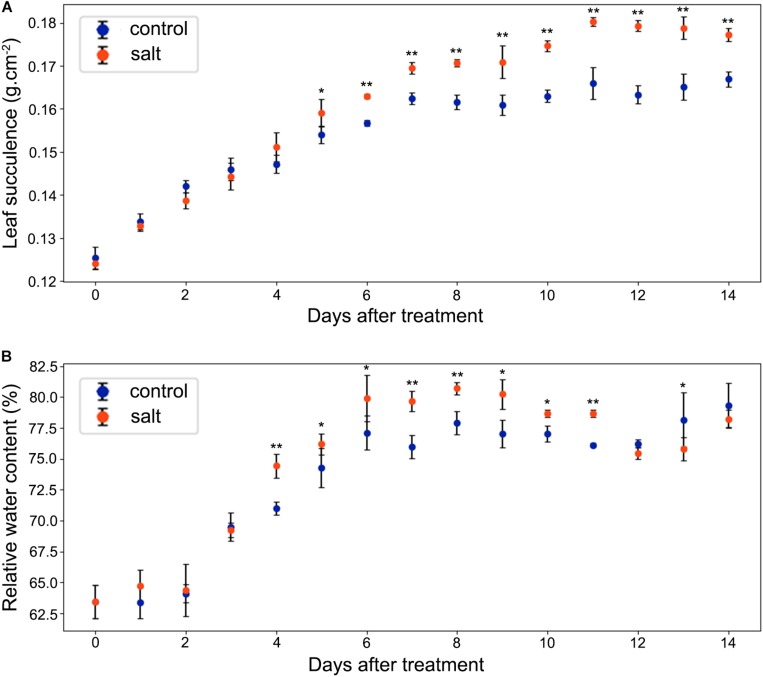
Changes in leaf succulence and relative water content (RWC) in the control and salt-treated *M. crystallinum* seedlings. **(A)** Leaf succulence. The blue dots represent the control group and the orange dots represent the salt-treated group. The error bar represents standard error. **(B)** RWC. The blue dots represent the control group and the orange dots represent the salt-treated group. The error bar represents standard error. An asterisk indicates a Student’s *t-*test (*p* < 0.05) and two asterisks indicate a Student’s *t-*test (*p* < 0.01). The data were obtained from four biological replicates.

As shown in Figure 4A, leaf succulence of both control and salt-treated *M. crystallinum* seedlings increased slowly from 0.12 to 0.16 g cm^–2^ during the beginning 5 days. In salt-treated *M. crystallinum*, leaf succulence was significantly higher than the control group after 5 days of the salt-treatment, and it increased to 0.18 g cm^–2^ after day 11. The RWC data showed *M. crystallinum* seedlings had an increase in water storage during the leaf growth from 62.5 to 80% (Figure 4B), which provides a strong support of the increasing leaf succulence of *M. crystallinum* at this developmental stage. Unlike the leaf succulence result, RWC of the salt-treated plants was significantly higher than control plants from day 4 to day 11, but it dropped to similar levels as control plants after day 12.

### MDA Content

**FIGURE 5:**
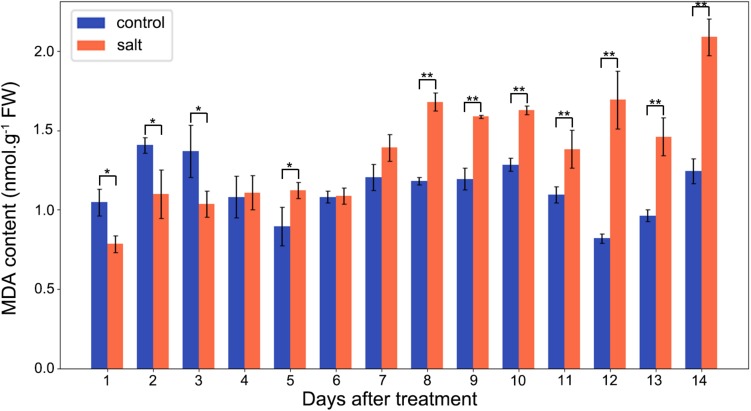
Changes in MDA contents in the control and salt-treated *M. crystallinum* seedlings. The blue dots represent the control group and the orange dots represent the salt-treated group. The error bar represents standard error. An asterisk indicates a Student’s *t-*test (*p* < 0.05) and two asterisks indicate a Student’s *t-*test (*p* < 0.01). The data were obtained from four biological replicates.

The MDA contents of *M. crystallinum* leaves were in a range of 0.8–1.5 nmol g^–1^ fresh weight in the control group. From day 7 after the salt treatment, the MDA contents began to show significant increases compared to the control group. Overall, the salt-treated group had higher MDA contents than the control group, and they were in a range of 1.4–2.1 nmol g^–1^ fresh weight after day 7 of the salt treatment (Figure 5).

## Discussion

*Mesembryanthemum crystallinum* is a well-known facultative CAM plant, which can change its stomatal movement pattern and gas exchange profile [e.g., from direct Rubisco-mediated CO_2_ assimilation to PEPC-mediated assimilation (Silvera et al., 2010; Winter and Holtum, 2014)]. Stomatal conductance mediated by guard cell circadian cycle needs to be synchronized with the mesophyll CAM cycle and the associated metabolite changes (Gallé et al., 2015; Males and Griffiths, 2017). It was reported that a relatively low nocturnal temperature may help to optimize the CAM activity (Yamori et al., 2014). To facilitate the C_3_ to CAM transition after salt treatment, we applied a consistent environment with relatively high diurnal temperature (26°C) and low nocturnal temperature (18°C). Under our conditions, the *M. crystallinum* seedlings showed normal C_3_ growth and development (Figure 1; Adams et al., 1998). When salt treatment was applied, the seedlings continue to grow at the similar rate as control for 6 days, and thereafter slowed down the growth and leaf expansion (Figures 1, 2). The phenotypic data reflect the shift from C_3_ to CAM, and the shift clearly compromised the seedling growth and development. How salt stress triggers the transition from C_3_ to CAM is still a mystery. Although it is challenging to differentiate salt stress responses from the specific responses leading to the C_3_ to CAM transition, studying the changes in diel patterns at molecular and physiological levels may provide important insights into the CAM initiation.

According to the net CO_2_ exchange data, *M. crystallinum* plants were determined to switch from C_3_ to CAM under drought stress and revert to C_3_ upon re-introduction of water to plants (Winter and Holtum, 2014). Our gas exchange data showed the transition from C_3_ to CAM under salt stress took place from 6 to 8 days after salt treatment and the CO_2_ exchange values were consistent with those reported in Winter and Holtum (2014) at both the C_3_ stage and CAM stage (Figure 3). Interestingly, leaf succulence and RWC increased in the salt-treatment group (Figure 4) before the gas exchange value had significant changes between the salt-treatment group and the control group (Figure 3A). These results seem to suggest succulence may be a prerequisite for the development of CAM photosynthesis (Bohnert and Cushman, 2000). During and after the C_3_ to CAM transition of the salt-treated seedlings, the leaf succulence maintained at higher levels than control seedlings. This result is consistent with the report that another facultative CAM plant *Ananas comosus* (L.) Merr. var MD-2 showed high leaf succulence after 4 weeks in CAM-inducing conditions (Aragón et al., 2013). Leaf succulence due to enlarged vacuoles contributes to not only malic acid storage, but also water storage under drought or salt stress. In addition, tightly packed large cells restrict CO_2_ efflux and enhance CO_2_ assimilation efficiency (Aragón et al., 2012, 2013). It should be noted that succulence and CAM are closely associated across the tree of life, although some CAM species (e.g., bromeliads) do not have succulent photosynthetic organs (De Santo et al., 1983; Ogburn and Edwards, 2013; Edwards, 2019).

In this study, we observed that significant changes in stomatal movement behavior occurred in the night of day 7 after the salt treatment (Figure 3B). This inverted stomatal movement behavior is essential for the nocturnal carbon fixation of CAM plants (Males and Griffiths, 2017; Edwards, 2019). Currently, there is little experimental data on the signaling pathways that control the inverse stomatal pattern in CAM plants. What drives the inversed stomatal behavior has been under debate. It was proposed that the nocturnal stomatal opening is mediated by the low CO_2_ concentration in the intercellular air space due to the PEPC activity, i.e., stomatal opening is driven by the nocturnal CO_2_ fixation (Cockburn, 1979; Griffiths et al., 2007; von Caemmerer and Griffiths, 2009). There are limited experimental data providing correlation but not direct cause and effect (Cockburn, 1979; Kebeish et al., 2012). For example, expression of a *Solanum tuberosum PEPC* under the control of a dark-induced promoter Din 10 in Arabidopsis resulted in greater stomatal conductance, respiration, and transpiration in dark-adapted leaves than wild-type plants (Kebeish et al., 2012). However, there is no evidence to imply the cause of stomatal movement is CO_2_. In fact, humidity should also be considered as humidity is generally high in the night (Meinzer, 2002). Other data appear to refute the role of CO_2_ in CAM induction. For example, in two facultative species, *Clusia pratensis* and *M. crystallinum* at C_3_ state, 100 or 800 ppm CO_2_ treatment during daytime showed no effect on nocturnal CO_2_ exchange, i.e., no CAM induction (Winter, 1979; Winter and Holtum, 2014). These data suggest that CO_2_ concentration may not be the CAM inducing factor. In addition, mesophyll-derived apoplastic malate was recently reported to link stomatal regulation with mesophyll cell metabolism (Medeiros et al., 2016).

High salinity can induce oxidative stress in plants (AbdElgawad et al., 2016). In this study, we measured MDA content as an indication of oxidative stress. Interestingly, MDA content did not significantly increase until day 8 after salt treatment, i.e., before the initiation of the transition from C_3_ to CAM photosynthesis (Figure 5). Previous studies indicated that oxidative stress could facilitate the switch from C_3_ to CAM (Aragón et al., 2012, 2013). After 8 days of salt treatment, the relatively high MDA content was maintained in the *M. crystallinum* seedlings, suggesting oxidative stress may be part of the CAM development and maintenance. However, it should be noted that the difference between the control group and the salt-treatment group was similar to some of the non-CAM halophytes (Ksouri et al., 2007; Amor et al., 2005), or significantly smaller than some non-halophytes (Liang, 1999; Sreenivasulu et al., 1999). Since reactive oxygen species and oxidative stress affect cellular redox state, and many biological processes including photosynthesis are regulated by redox, future studies focusing on redox regulation [e.g., using redox proteomics and metabolomics (David et al., 2019; Yu et al., 2020)] can be expected to reveal molecular mechanisms underlying the C_3_ to CAM transition in *M. crystallinum* plants.

## Conclusion

*Mesembryanthemum crystallinum* is a facultative CAM plant, which can switch from C_3_ photosynthesis to CAM photosynthesis under salt stress treatment. Based on a combination of phenotypic and physiological measurements (including leaf area, gas exchange, stomatal aperture, leaf succulence, RWC and MDA contents), we found the critical transition time for *M. crystallinum* seedlings to shift from C_3_ to CAM photosynthesis is between 6 and 8 days of salt treatment. The quick transition to CAM photosynthesis is important for the seedlings to establish tolerance to environmental stresses with CAM characteristics, such as high WUE and inverted stomatal behavior. Our study has laid a foundation for further experiments to determine the molecular switches underlying the rapid C_3_ to CAM transition, and thereby engineering CAM into C_3_ crops for enhanced WUE and stress tolerance.

## Data Availability Statement

All datasets generated for this study are included in the article/Supplementary Material.

## Author Contributions

QG, JT, and SC conceived and designed the research. QG, BT, and TK performed the experiments. QG, BT, and SC analyzed the data and wrote the manuscript draft. JT and SC finalized the manuscript for submission. All authors approved the manuscript.

## Conflict of Interest

The authors declare that the research was conducted in the absence of any commercial or financial relationships that could be construed as a potential conflict of interest.

## Footnotes

1 https://imagej.nih.gov/ij/

## Supplementary Material

FIGURE S1 Development of a method for measuring leaf area. **(A)** Positioning of the plant and 1 cm^2^ reference spot. **(B)** Original image taken by a digital camera. **(C)** Border outline of the leaves, as a quality control for the pixel selection. **(D)** Total reference pixels detected in the image, and white pixels represent the reference pixels. **(E)** Total leaf pixels detected in the plant image; light yellow pixels represent the pixels detected in green.

FIGURE S2 Stomatal aperture changes in day and night during the C_3_ to CAM transition of *M. crystallinum* seedlings in the control and the salt groups. The data were collected at 4 am representing night and 4 pm representing day.

FILE S1 A python script for detecting green pixels as leaf pixels, with black pixels as reference pixels to calculate the leaf area by green pixels ^∗^ actual reference area divided by the reference pixels.
