# Supplementary figures and images for: Physiological Changes in Mesembryanthemum crystallinum During the C3 to CAM Transition Induced by Salt Stress
Source: Front Plant Sci. 2020 Mar 17;11:283. doi: 10.3389/fpls.2020.00283 (PMC7090145; doi:10.3389/fpls.2020.00283)

A

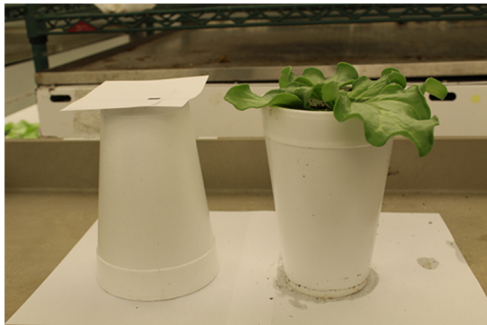

B

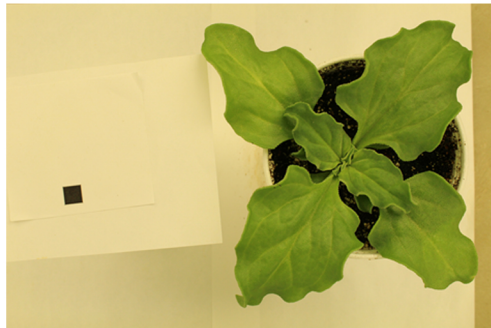

C

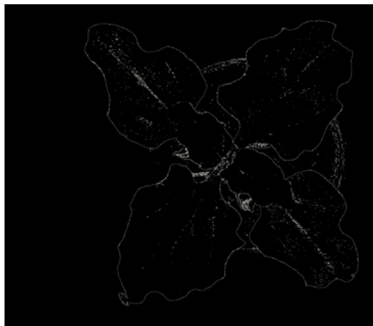

D

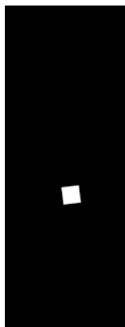

E

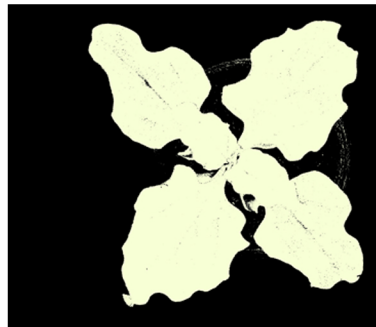

Supplement: FIGURE S1 — Development of a method for measuring leaf area. (A) Positioning of the plant and 1 cm2 reference spot. (B) Original image taken by a digital camera. (C) Border outline of the leaves, as a quality control for the pixel selection. (D) Total reference pixels detected in the image, and white pixels represent the reference pixels. (E) Total leaf pixels detected in the plant image; light yellow pixels represent the pixels detected in green. [file Data_Sheet_1.PDF]

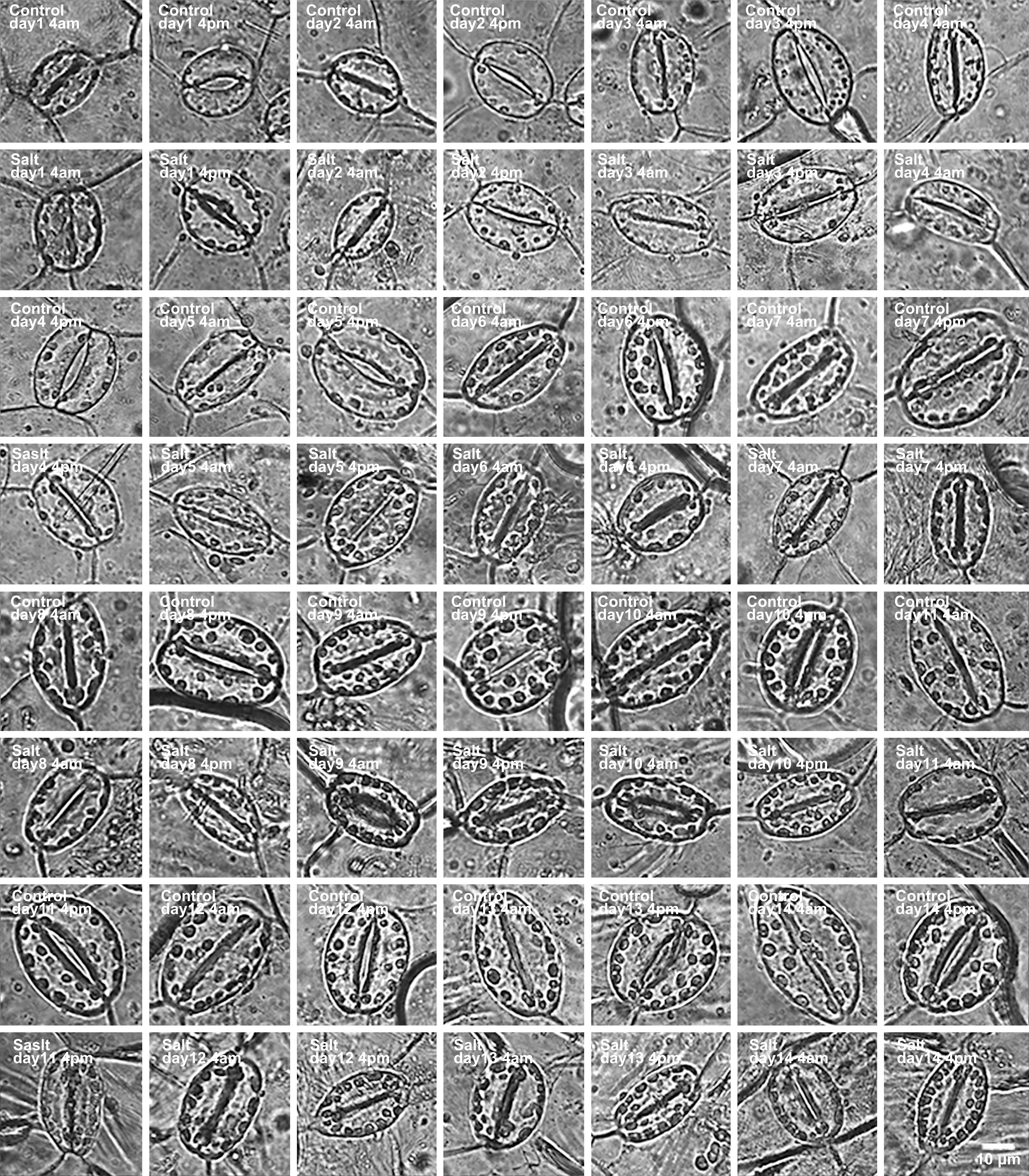

Supplement: FIGURE S2 — Stomatal aperture changes in day and night during the C3 to CAM transition of M. crystallinum seedlings in the control and the salt groups. The data were collected at 4 am representing night and 4 pm representing day. [file Data_Sheet_2.PDF]
