# Supplementary material for: Physiological Changes in Mesembryanthemum crystallinum During the C3 to CAM Transition Induced by Salt Stress
Source: Front Plant Sci. 2020 Mar 17;11:283. doi: 10.3389/fpls.2020.00283 (PMC7090145; doi:10.3389/fpls.2020.00283)
Supplement: FILE S1 — A python script for detecting green pixels as leaf pixels, with black pixels as reference pixels to calculate the leaf area by green pixels ∗ actual reference area divided by the reference pixels. [file Table_1.DOCX]

# coding=utf-8

# to use this script, please copy the code to an .py file without formats and coding.

from PIL import ImageFilter

from PIL import Image

import sys

__author__ = 'Qijie Guan'

# Usage: python3 measure_leaf_area.py [path of figure]

# Convert RGB file to HSV

im_sample_name = sys.argv[1]

im_raw = Image.open(im_sample_name)

width_raw = im_raw.size[0]

height_raw = im_raw.size[1]

im = im_raw.crop((width_raw/4,0,width_raw,height_raw))

width = im.size[0]

height = im.size[1]

im = im.convert('HSV')

im = im.filter(ImageFilter.SHARPEN)

im_pixel = im.load()

count_leaf_area = 0

for i in range(width):

    for j in range(height):

        # detect green pixels here

        if im_pixel[i,j][0]>=40 and im_pixel[i,j][0]<=78 and im_pixel[i,j][2]>=80 and im_pixel[i,j][2]<=230:

            im_pixel[i,j] = (50, 43, 255)

            count_leaf_area += 1

        else:

            im_pixel[i,j] = (0,0,0)

# Convert HSV file to RGB for outputing

im = im.convert('RGB')

im.save(im_sample_name+'.measured_area.jpg', 'JPEG' )

im_find_edges = im.filter(ImageFilter.FIND_EDGES)

im_find_edges.save(im_sample_name+'.border.jpg','JPEG')

#print(count_leaf_area)

im_reference = im_raw.crop((0, 0, width_raw/4,height_raw))

width_r = im_reference.size[0]

height_r = im_reference.size[1]

im_r = im_reference.filter(ImageFilter.SHARPEN)

im_r = im_reference.convert('HSV')

im_r_pixel = im_r.load()

count_reference = 0

for i in range(width_r):

    for j in range(height_r):

        # detect black pixels here

        if im_r_pixel[i,j][2] <= 150:

            im_r_pixel[i,j] = (180,0,255)

            count_reference += 1

        else:

            im_r_pixel[i,j] = (0,0,0)

im_r = im_r.convert('RGB')

im_r.save(im_sample_name+'.refrence.jpg','JPEG')

print(count_leaf_area/count_reference)

fOUT = open(im_sample_name+'.area.txt', 'w')

fOUT.write(str(count_leaf_area/count_reference))

fOUT.close()
